# *Sida tuberculata* (Malvaceae): a study based on development of extractive system and *in silico* and *in vitro* properties

**DOI:** 10.1590/1414-431X20165282

**Published:** 2016-07-11

**Authors:** H.S. da Rosa, A.C.F. Salgueiro, A.Z.C. Colpo, F.R. Paula, A.S.L. Mendez, V. Folmer

**Affiliations:** 1 Laboratório de Bioquímica e Toxicologia de Produtos Naturais e Sintéticos, Universidade Federal do Pampa, Uruguaiana, RS, Brasil; 2 Laboratório de Desenvolvimento e Controle de Qualidade em Medicamentos, Universidade Federal do Pampa, Uruguaiana, RS, Brasil; 3 Programa de Pós-Graduação em Bioquímica, Universidade Federal do Pampa, Uruguaiana, RS, Brasil; 4 Faculdade de Farmácia, Universidade Federal do Rio Grande do Sul, Porto Alegre, RS, Brasil

**Keywords:** Sida tuberculata, 20-hydroxyecdysone, Toxicity prediction, Antioxidant

## Abstract

*Sida tuberculata* (Malvaceae) is a medicinal plant traditionally used in Brazil as an antimicrobial and anti-inflammatory agent. Here, we aimed to investigate the different extractive techniques on phytochemical parameters, as well as to evaluate the toxicity and antioxidant capacity of *S. tuberculata* extracts using *in silico* and *in vitro* models. Therefore, in order to determine the dry residue content and the main compound 20-hydroxyecdysone (20E) concentration, extracts from leaves and roots were prepared testing ethanol and water in different proportions. Extracts were then assessed by *Artemia salina* lethality test, and toxicity prediction of 20E was estimated. Antioxidant activity was performed by DPPH and ABTS radical scavenger assays, ferric reducing power assay, nitrogen derivative scavenger, deoxyribose degradation, and TBARS assays. HPLC evaluation detected 20E as main compound in leaves and roots. Percolation method showed the highest concentrations of 20E (0.134 and 0.096 mg/mL of extract for leaves and roots, respectively). All crude extracts presented low toxic potential on *A. salina* (LD_50_ >1000 µg/mL). The computational evaluation of 20E showed a low toxicity prediction. For *in vitro* antioxidant tests, hydroethanolic extracts of leaves were most effective compared to roots. In addition, hydroethanolic extracts presented a higher IC_50_ antioxidant than aqueous extracts. TBARS formation was prevented by leaves hydroethanolic extract from 0.015 and 0.03 mg/mL and for roots from 0.03 and 0.3 mg/mL on egg yolk and rat tissue, respectively (P<0.05). These findings suggest that *S. tuberculata* extracts are a considerable source of ecdysteroids and possesses a significant antioxidant property with low toxic potential.

## Introduction

*Sida* species are widespread around the world, occurring predominantly in the tropics, particularly in South America. Some species of this genus has been employed in traditional medicine for a long time, such as *S. rhombifolia*, *S. acuta* and *S. cordifolia* (1,2). In Brazil, *Sida* species are used in folk medicine for treatment of stomatitis, blenorrhea, asthmatic bronchitis and other inflammatory processes (3,4). Among the several species of this genus is *S. tuberculata* (Malvaceae), a medicinal plant widely distributed in South Brazil. Traditionally, leaves and roots of this species have been used as anti-inflammatory, hypoglycemic and antimicrobial agents.

Previous studies with different extracts and isolated compounds of this genus have described important biologic effects. Aqueous extracts from *S. cordifolia* reduced the damage caused by rotenone and presented a therapeutic action in Parkinson's disease (5). *S. acuta* revealed a significant hepatoprotective effect against liver damage induced by paracetamol overdose (6). Leaf extracts of *S. rhomboidea* demonstrated a significant cardiovascular protective effect (7). The anti-inflammatory activity also was investigated for *S. tiagii* extracts, which presented similar results to the standard drugs tested (8). Our group previously found a significant antimicrobial effect of *S. tuberculata* extracts against *C. krusei* strain (9).

Chemical investigations of *Sida* spp. have indicated the presence of a wide variety of compounds. Among the main classes of chemicals detected, ecdysteroids (10), alkaloids (11) and flavonoids (12) are predominant. Within the ecdysteroids class, polyhydroxylated ketosteroids and its derivatives are the most frequent (13,14). Ecdysteroids are produced primarily in arthropods and plants, but are also present in fungi, and even in marine sponges (15). Interesting observations on the potential importance of ecdysteroids have justified studies on function and biological properties of this class.

Recently, our research group identified, among others, 20-hydroxyecdysone (20E), a major ecdysteroid in *S. tuberculata*, as well as flavonoids and alkaloids (9). Thus, considering our interest about *S. tuberculata* biological properties, in the current work, different extracts were evaluated based on phytochemical parameters and assessed for their toxicological and antioxidant potential.

## Material and Methods

### Plant material

The whole plant was collected in Uruguaiana (Rio Grande do Sul, Brazil), a city located at the western border with Argentina. A specimen was identified and a voucher (*Sida tuberculata* R.E. Fries; ICN 167493) was deposited at ICN Herbarium (Instituto de Biociências, Universidade Federal do Rio Grande do Sul, Brazil).

### Extract obtainment

Initially, the plant was separated into leaves and roots. Each material was submitted to drying at 40°C, reduced to a powder and submitted to extraction by maceration, reflux, and percolation techniques. Aiming to evaluate the most adequate system, three ethanol concentrations were tested on extraction: 20, 30 and 40% (v/v) for leaves and 50, 70 and 90% (v/v) for roots. This choice was based on the plant’s tissue rigidity. In all cases, the plant:solvent proportion was standardized at 1:10 (w/v). Aqueous infusions (tea) were also prepared for toxicity and antioxidant assays according to methodologies described below.

### Dry residue determination

Dry residue assay was performed according to Brazilian Pharmacopoeia (16). Briefly, 2.0 mL of each extract were submitted to drying at 105°C until a residual mass correspondent to the concentration of dried extractives was obtained. All developed extracts were assayed in triplicate.

### Chromatographic analysis

Extract samples were evaluated by a high performance liquid chromatograph coupled with diode array detection (HPLC-DAD) (9). Chromatography analysis was performed with a Prominence liquid chromatograph (Shimadzu, Japan), equipped with a binary pump LC-20AD with SIL-10AF auto sampler and SPDM10A PDA detector. Mobile phase consisted of (A) 0.05% phosphoric acid in water and (B) acetonitrile, prepared daily, filtered through a 0.45-mm membrane filter (Millipore, Germany) and sonicated before use. The separation was accomplished using a Phenomenex Luna C-18(2) column (250×4.6 mm, 5 µm) with a gradient elution protocol of 0.01-23 min, 10-40% solvent B; 23.01-40 min, 10% solvent B, at a flow rate of 0.8 mL/min. Injection volume was 20 µL and DAD detector was operated at 250 nm.

*20-hydroxyecdysone monitoring*. Aiming to determine the concentration of the major compound 20E in extract, five concentrations (10, 50, 100, 200 and 500 µg/mL) of standard solution (20E, Sigma Aldrich, USA, 93% purity) were prepared in methanol. Chromatographic injections were made in triplicate. All solutions were freshly prepared and filtered through a 0.45-mm membrane filter (Millipore), prior to analysis.

### Computational prediction

Aiming to identify possible nutraceuticals, we evaluated the predictive toxicity of 20E. For this, we analyzed the risks of damages, such as genotoxic damage, endocrine disruption, irritation and hERG (the human ether-è-go-go-related gene) inhibition. Data were generated on-line using ADME-Tox web server software, Advanced Chemistry Development, Inc. (ACD/Labs, ACD/Percepta Platform, version 12.01, Canada, www.acdlabs.com, 2013) (17), which predicts the fragments that could lead to possible toxic effects.

In addition, the 20E was subjected to the drug-likeness evaluation and drug-score profiles using the Osiris Property Explorer program available on the web [http://www.organic-chemistry.org/prog/peo] (18), comparing it with a reference substance.

### *Artemia salina* assay

Based in dry residue and HPLC analysis, we selected extracts (ethanol 70 and 40% for roots and leaves, respectively) to be used in this test. Extracts were concentrated and ethanol was evaporated.

The test was performed according to Meyer et al. (19) with minor modifications. Briefly, *A. salina* eggs were incubated in seawater with 3% NaCl at room temperature for 24 h. After, *A. saline* larvae (10 approximately) were transferred to ELISA plate wells containing different extract concentrations (100-1000 μg/mL) prepared by diluting the extract in 10 mL of the artificial saline solution. For control, larvae were incubated with seawater only. Plates were maintained at 28±1°C for 24 h and the survival rate (%) was counted for lethal dose 50% (LD_50_) determination. Three independent experiments were performed.

### Determination of antioxidant capacity

For antioxidant activity protocols, solutions obtained by percolation with ethanol 70 and 40% for roots and leaves, respectively, were selected. The aqueous extracts were also evaluated with these protocols. For all assays described below, the samples were diluted to obtain a concentration range of 0.003-0.3 mg/mL. It is important to emphasize that the color controls were used for all extracts, avoiding probable interference of extracts color in results. Moreover, except for thiobarbituric acid reactive substances (TBARS) protocols in animal tissue, results are expressed in half maximal inhibitory concentration (IC_50_).

*DPPH• assay*. Antiradical activity of *S. tuberculata* extracts was determined using the DPPH^•^ method (20). Different concentrations of extracts were added to DPPH^•^ solution. After 30 min of incubation at room temperature, the reduction in the number of free radicals was measured by reading the absorbance at 517 nm. Values are reported in IC_50_ based on percentage of inhibition of DPPH^•^ absorbance in relation to the control values without extracts.

*ABTS•+ scavenger activity*. ABTS^•+^ radical cation (21) was obtained by the reaction between the ABTS solution with the K_2_S_2_O_8_ (140 mM) solution for 12-16 h in the dark at room temperature. Antioxidant assay was performed by incubation of extract samples and ABTS^•+^ (final volume of 1.5 mL) during 6 min in the dark. The absorbance was measured at 734 nm. Ethyl alcohol was used as blank to calibrate the spectrophotometer.

*Ferric reducing potential assay (FRAP)*. The ferric reducing power of *S. tuberculata* extracts was determined using a modified version of the FRAP assay (based on the chemical reduction of Fe^3+^ to Fe^2+^) (22). Briefly, aliquots of the extract were added to freshly prepared and pre-warmed (37°C) FRAP reagent and incubated at 37°C for 30 min. Reduction was monitored by measuring the change of absorbance at 593 nm.

*Nitrogen derivative species scavenging activity*. According to Marcocci et al. (23), the assay is based on the reaction of nitric oxide radical (NO^•^) produced by sodium nitroprusside in aqueous solution at physiological pH 7.2. Under aerobic conditions, NO^•^ reacts with oxygen to produce nitrogen derivative products (i.e., nitrate and nitrite) which can be determined using Griess reagent. Values report the percentage of nitrite reaction inhibition with Griess reagent depicted by the *S. tuberculata* extracts as an index of the NO^•^ scavenging activity.

*Deoxyribose assay*. This assay was performed in accordance with modifications proposed by Puntel et al. (24). Here, hydroxyl radicals were generated by Fenton reaction. Antioxidant capacity was evaluated by the extract's ability to neutralize hydroxyl radicals. Results are reported in IC_50_ based on percentage of inhibition.

*Lipid peroxidation assay*. Using egg-yolk homogenates, a modified TBARS protocol was employed to measure the formed lipid peroxide (25). Briefly, egg yolk was homogenized and mixed with *S. tuberculata* extracts and FeSO_4_. This mixture was incubated at 37°C for 60 min, and used in the TBARS assay. Values are reported in equivalents of malondialdehyde (MDA) generated by lipid peroxidation and corrected by mg of tissue.

*TBARS in brain and liver of rats*. A total of 4 adult male rats (Wistar) were maintained and used in accordance with guidelines of the Committee on Care and Use of Experimental Animal Resources (Protocol approved #001/2012, UNIPAMPA). The animals were sacrificed by decapitation and the brain and liver were removed, quickly homogenized in NaCl (150 mM) and kept on ice. TBARS content was determined as described by Ohkawa et al. (26), using a standard curve of MDA. Briefly, after homogenization, samples were centrifuged at 4000 *g* at 4°C for 10 min to yield a low speed supernatant fraction (S1). The obtained S1 was used for basal and/or pro-oxidants (FeSO_4_) induced lipid peroxidation. This mixture was incubated at 37°C for 60 min, and after used in the TBARS assay. Values are reported in nmol of MDA generated by lipid peroxidation and corrected by protein content.

### Statistical analysis

Data are reported as means±SD for at least three independent determinations for each experimental step. Statistical differences between groups were determined by two-way ANOVA with the Tukey's post tests. Values of P≤0.05 were considered the limit for significance.

## Results

### Dry residue

Results obtained from dry residue assay are described in Table 1. The higher yield of dry content occurred when the extracts were prepared by percolation. In terms of alcoholic concentration, the most efficient solvents were hydroethanolic solutions at 40% for leaves and at 70% for roots, when the dry residue parameter was considered alone.

**Table:**
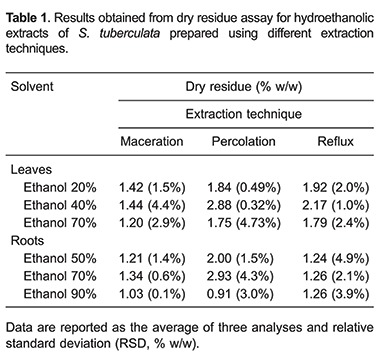

### Chromatographic analysis

HPLC analyses were performed evaluating *S. tuberculata* extracts obtained from maceration, percolation and reflux. In leaves, two major peaks were identified, with retention times (Rt) of approximately 17.2 (peak 1) and 18.0 min (peak 2). The most representative ecdysteroid (20E) present in the leaves (peak 1) was also accompanied by significant amounts of another phenolic compound, a kaempferol derivative (peak 2). In roots, 20E was detected as the major compound.

**Figure 1:**
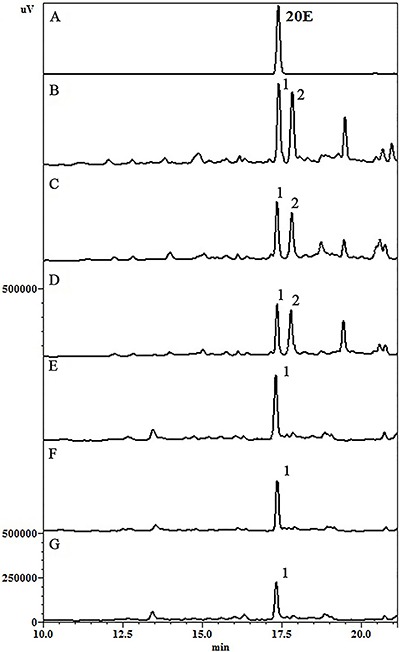
Comparative chromatogram of *S. tuberculata* extracts, using 40% ethanol for leaves and 70% ethanol for roots. *A*, reference substance of 100 µg/mL 20-hydroxyecdysone (20E). *B*, *C*, and *D*, leaf hydroethanolic extract obtained by percolation, reflux and maceration, respectively. *E*, *F*, and *G*, root hydroethanolic extract obtained by percolation, reflux and maceration, respectively. Peak 1: 20E, Peak 2: Kaempferol derivative, acquired at 250 nm.

In order to evaluate extraction efficiencies, the peak areas of 20E were considered. Data showed that percolation was the most effective technique followed by reflux and maceration (Figure 1). In addition, results revealed that 20E concentration was greater in leaves than roots extracts, 0.134 and 0.096 mg/mL, respectively (Table 2).

**Table:**
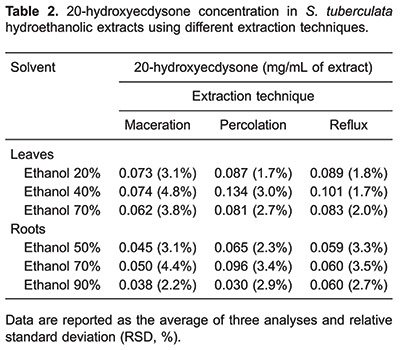

### *In silico* predictions

The 20E compound was submitted to computational prediction of toxic effects using ACD/Labs. This effect was evaluated by the probability of producing a positive Ames Test outcome (Ames test against *Salmonella typhimurium* TA97a, TA98, TA100, TA102, TA104, TA1535, TA1537 and TA1538), a positive endocrine disruptor test outcome, hERG inhibition and irritant effects. 20E showed a low probability to cause toxic effects in all the parameters evaluated (Table 3). Thus, these data suggest that 20E may be non-genotoxic, non-reproductive-system toxic, non-cardiotoxic and non-irritant. The software also predicted that 20E does not present hazardous fragments, which are interconnected with mutagenic effects.

The Osiris Property Explorer calculated the drug-likeness and drug-score characteristics based in the list of all available fragments from 3300 traded drugs as well as 15,000 commercially available chemicals (Fluka, Germany). In this work, Osiris results showed that 20E had a positive drug-likeness (0.62) and drug-score (0.2) values (Table 4). The 20E values were greater than those of α-tocopherol (-6.2 drug-likeness; 0.11 drug-score, respectively), and greater than drug-likeness of ascorbic acid (0.02).

**Table:**
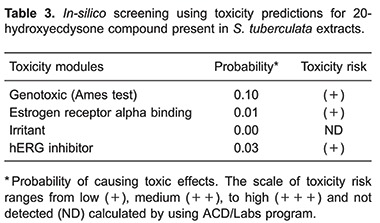

**Table:**
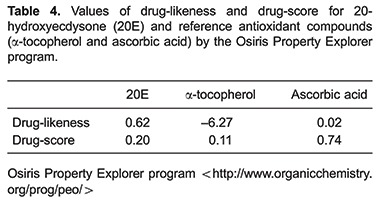

### *Artemia salina* toxicity

Results of *A. salina* toxicity are shown in Table 5. *S. tuberculata* presented low toxicity to *A. salina* larvae (LD_50_ >1000 µg/mL) for both leaves and roots extracts.

**Table:**
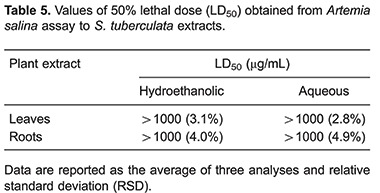

### Antioxidant assays

In the DPPH^•^ assay, the hydroethanolic extracts from leaves showed the most effective result with IC_50_ activity at 0.116 mg/mL followed by hydroethanolic root with IC_50_ of 0.142 mg/mL (Table 6). Aqueous extracts showed the lowest antioxidant capacity.

**Table:**
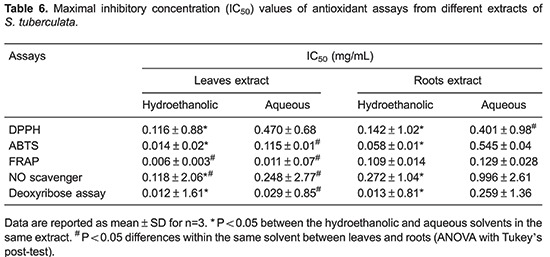

In the ABTS⋅^+^ assay, IC_50_ values ranged from 0.014 (hydroethanolic leaf) to 0.545 mg/mL (aqueous root). Compared to DPPH^•^ assay, hydroethanolic leaves had the highest potential scavenger, approximately 10-fold. In addition, hydroethanolic leaf extract possessed the highest ABTS^•+^ inhibition followed by root extract.

In the FRAP protocol, the oxidative form of iron (Fe^+3^) is converted to ferrous (Fe^+2^) by antioxidant compounds. Extracts of *S. tuberculata* expressed great reducing activity. As shown in Table 6, hydroethanolic leaf extract had the most pronounced effect of all assessed protocols (IC_50_=0.006 mg/mL). Similarly, aqueous extracts of leaves were also potent in FRAP activity (IC_50_=0.011 mg/mL).

The nitrogen reactive species scavenger test illustrates percentage inhibition of nitrogen reactive species by extracts from leaves and roots of *S. tuberculata*. The IC_50_ value of hydroethanolic and aqueous extracts ranged from 0.118 and 0.996 mg/mL (Table 6). Both extracts, leaves and roots, showed a significant (P<0.05) scavenging activity. However, extracts of leaves had more scavenger property than root extracts.

In deoxyribose degradation assays, extracts presented a potent scavenger activity of hydroxyl radical, one of the most aggressive oxidants formed from Fenton reactions. In this regard, *S. tuberculata* extracts significantly inhibited the oxidation of deoxyribose in low concentrations (Table 6). Overall, hydroethanolic extracts showed stronger inhibition activity than aqueous extracts.

**Figure 2:**
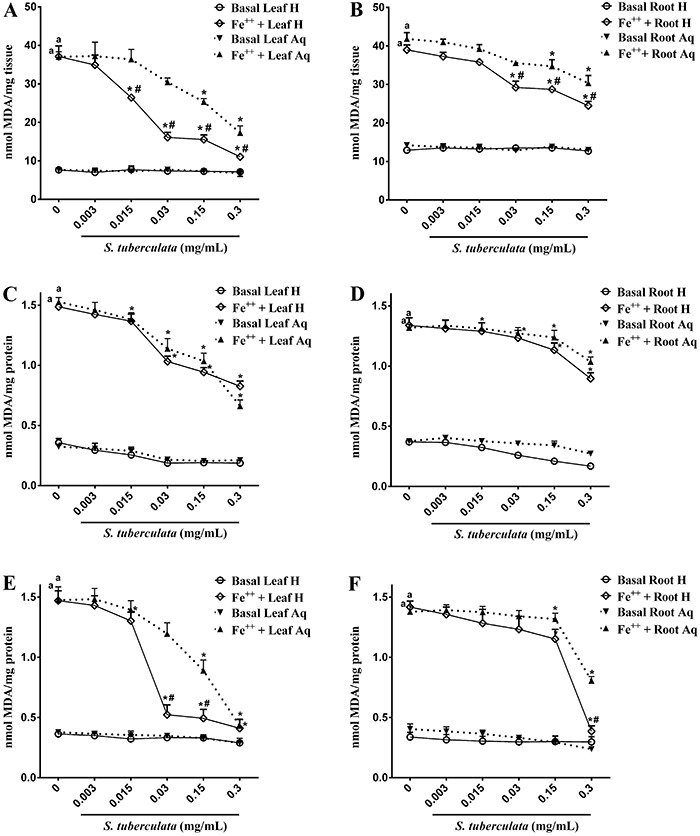
Effects of *S. tuberculata* extracts on TBARS production in egg yolk lipids (*A*, *B*), brain (*C*, *D*) and liver (*E*, *F*) of rats. Hydroethanolic leaf extract (Leaf H), aqueous leaf extract (Leaf Aq), hydroethanolic root extract (Root H), and aqueous root extract (Root Aq) were evaluated. Results are reported as nmol of MDA per mg of tissue (*A*, *B*) or mg of protein (*C*-*F*) for (n=3). ^a^P≤0.05 compared to basal not induced (i.e., zero concentration); *P≤0.05 compared to basal in the same group (i.e., zero concentration); ^#^P≤0.05 compared to the same concentration of the other extracts evaluated (two-way ANOVA with the Tukey's post tests).

Analyses of lipid peroxidation from egg yolks (Figure 2A and B) showed that both extracts of *S. tuberculata* inhibited lipid peroxidation. Hydroethanolic extracts exhibited significant inhibition from 0.015 and 0.03 mg/mL for leaves and roots, respectively (P≤0.05). Aqueous extracts showed significance from 0.15 mg/mL for both parts of plants. Comparing leaves and roots, the leaves had greater antioxidant activity than roots.

Figure 2C and D shows the effects of *S. tuberculata* extracts on lipid peroxidation caused by Fe^+2^ in rat brain homogenates. The iron concentration tested (0.01 mM) induced a significant oxidative damage (P≤0.05). We observed that hydroethanolic extracts from leaves and roots presented a significant decrease on TBARS formation (P≤0.05) from 0.015 and 0.03 mg/mL concentrations, respectively. Aqueous extracts showed a significant reducing effect from 0.03 and 0.3 mg/mL concentrations for leaves and roots, respectively.

In liver tissue, all extracts of *S. tuberculata* inhibited TBARS production (Figure 2E and F). However, the hydroethanolic extracts exerted a more pronounced effect. At concentrations of 0.03 and 0.3 mg/mL, hydroethanolic extracts of leaves and roots inhibited lipid peroxidation to almost baseline levels.

## Discussion

The present study describes an investigation about the medicinal plant *S. tuberculata* from Brazilian Pampa biome. We evaluated the dry residue and the concentration of the major compound (20E) with different extraction techniques applied on *S. tuberculata*. The data were used to select the extracts to be applied in toxicity and antioxidant assays. This method has a central role in obtaining products with constant composition and reproducible biological properties.

Considering the parameters evaluated for leaves and roots, percolation was the most effective technique. Results showed that percolation method improved the dry residue and 20E concentration. This finding may be related to the technique’s exhaustive extraction and solvent renewal. Differently from maceration and reflux, in percolation the solvent remains 1 h in contact with the sample and then elutes through the column more than once with fresh solvent. Moreover, percolation methodology does not involve heating, an important aspect taking into account the thermolability of some phytoconstituents.

Our chromatographic analysis confirmed 20E as the main metabolite in leaves and roots. Moreover, a kaempferol derivative was detected only in leaves. This finding is in accordance with a previous phytochemical study by our group (9). In addition, we observed the presence of more metabolites in leaves than in roots. This result may be partially explained by the sunlight influence on biosynthesis of some compounds such as flavonoids (27).

**Figure 3:**
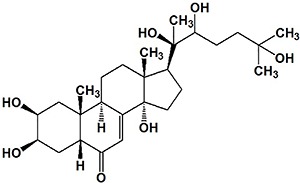
Representation of 20-hydroxyecdysone molecular structure (C_27_H_44_O_7_). PubChem CID: 5459840.

The major identified compound (20E) belongs to the ecdysteroids class. It has a steroidal nucleus and a polyhydroxylated chain (Figure 3). Ecdysteroids or "phytoecdysteroids" are the plant analogues of insect growth hormones. Their function in plants is unclear; however, they may be involved in the deterrence of invertebrate predators by acting as antifeed-systems, or yet by interfering in the ingestion of phytophagous insects (16). In mammals, 20E has demonstrated therapeutic properties including memory improvement, reduction of lipid storage and anabolic effects (28–30).

The predictive Ames test used here is performed worldwide as an initial screening to determine genotoxic properties of new chemical entities to be used by the pharmaceutical industry. It is a quick test, based on bacterial reverse mutation performed on various bacterial strains. The genotoxicity predicted by the Ames Test is based on an iterative model built using structural toxic fragments from a database as descriptors. 20E showed a low probability for genotoxicity (10%).

The endocrine disruptor test is associated with the binding of compounds to alpha estrogen receptor, which may be linked to reproductive toxicity and cancers (31). The 20E was classified as binder/non-binder, due to their relative binding affinities (RBA) compared to a reference ligand in the ACD/Labs database (17). Two cut-offs were used: LogRBA ≥3 ("general binding"), and LogRBA >0 ("strong binding") and 20E presented a 1% probability to be a LogRBA ≥3. This measurement is important since the 20E has a steroidal structure and may interact with *in vivo* hormonal receptors.

Another assessed toxic effect was the hERG inhibition, which is an ion channel that, when inhibited, is related to cardiovascular damage (32). It is essential to investigate any chemical entity for this potential cardiotoxic effect. A large number of drugs have been withdrawn from clinical trials or from the market due to the fatalities associated to hERG inhibition. The hERG predictive inhibition for 20E was 3%. Similarly, irritant properties related to skin and eye tissues were evaluated, and non-toxic results were found. The low toxic probability of 20E, may be in part due to the absence of hazardous fragments (predictive data), which are known toxic agents.

The *A. salina* toxicity assay is practical, inexpensive, simple, reliable and an important tool in routine plant toxicity screening. Our data showed very low toxicity of hydroethanolic and aqueous extracts from leaves and roots. As the test implies the presence of cytotoxic constituents, as initial screening, our results indicate that aqueous extracts used as folk medicine have a low risk of acute toxicity. However, further studies are necessary to establish the toxicological endpoints at systemic level.

Since this work proposed to investigate the antioxidant potential of *S. tuberculata* extracts, we evaluated the overall potential of its major compound to be qualified as a nutraceuticals product and, therefore, be available in the market. Thus, the compound 20E was subjected to an *in silico* screening to evaluate its theoretical drug-likeness and drug-score in comparison with antioxidants references, ascorbic acid and α-tocopherol. The positive values of drug-likeness indicated that 20E contains fragments as good as the references. These results increase the possibility of establishing the therapeutic actions of these fragments, known as pharmacophores, and of this compound becoming a possible nutraceuticals product.

The assessment of antioxidant activity was applied for 40% (leaves) and 70% (roots) hydroethanolic extracts, obtained by percolation technique. It is important to emphasize that the best extractive system was defined observing the results of all evaluated parameters, and trying to maintain the stability of metabolites. Aqueous infusions were assayed, with the purpose of evaluating the method usually employed by the population in preparing home remedies.

The antioxidant capacity of an extract or compound can be analyzed by several assays with different mechanisms (33). Generally, the chemical reaction involved in antioxidant *in vitro* assays fall into two categories: hydrogen atom transfer (HAT) assays, which use a competitive reaction between an antioxidant and a substrate, where both compete for peroxyl radicals thermally generated, and single electron transfer (ET) reaction assays, that measure the potential of an antioxidant to reduce an oxidant, which changes color when reduced. All these elements, advantages and limitations, need to be considered when evaluating and selecting a potential antioxidant.

In view of the above comments, we used six different methods to evaluate the antioxidant capacity of *S. tuberculata* extracts: DPPH^•^, ABTS^•+^, NO and FRAP based in ET assays, and deoxyribose and TBARS, which are based in HAT. Results indicated that both leaves and roots extracts, could act by ET and HAT mechanisms. Moreover, data showed that hydroethanolic extracts present a better antioxidant potential than aqueous extracts. This fact may be related with a greater extraction ability of ethanol than water alone. Yea et al. (34), evaluating the effect of different solvents on phenolic content, found a higher extraction capacity in aqueous alcohols than water.

We also observed that leaves presented higher scavenger properties than roots. This finding probably occurred due to the diversity of phytoconstituents present in leaves. In fact, our analysis identified a kaempferol derivative detected only in the leaves. In this context, phenolic compounds are known for its antioxidant properties, such as free radical scavenging and chelation of metal ions. Therefore, the presence of phenolic compounds may explain the notable antioxidant activity in leaves. Moreover, concomitant occurrence of phenols and ecdysteroids may improve antioxidant potential by synergistic effects (35). However, it is not possible to know precisely if ecdysteroid class compounds exert antioxidant effects.

Our results for DPPH^•^, ABTS^•+^, FRAP and NO assays are agreement with Pawar et al. (36) and Shah et al. (37), who reported a great antioxidant activity for *S. cordifolia* and *S. cordata*. It should be noted that the effect of *S. tuberculata* detected with Griess reaction may be due to scavenger activity of extracts for nitrogen derivative species, i.e., NO_2_, N_2_O_3_, N_2_O_4_, peroxynitrite (ONOO^-^) or even for different redox forms, such as nitrosonium (NO^+^) and nitroxyl anion (NO^-^) generated or interconverted from nitric oxide under physiological conditions. Therefore, the antioxidant activity is an important property, since it prevents the formation of deleterious oxidants that can react with biological molecules, particularly oxidizing iron/sulfur centers, zinc fingers, and protein thiols, which plays a relevant role in cardiovascular and neurological diseases (23,38).

In this context, it is known that iron plays a significant role in noxious oxygen species production. Iron initiates a chain of reactions leading to lipid peroxidation and consequent cellular damage. Our data showed a protective effect of all extracts of *S. tuberculata* against oxidative damage by deoxyribose and TBARS assays.

One possible protection mechanism against lipid peroxidation damage may be related to Fe^2+^ chelating activity. In this case, the extract binds to metal preventing it to interact with H_2_O_2_ avoiding hydroxyl radical (OH^•^) generation and consequently the damage. In other words, chelating compounds may decrease metal bioavailability inhibiting its participation in OH^•^ generation by the Fenton reaction (39,40). *S. tuberculata* extracts presented a significant Fe^2+^ chelating activity (data not shown), which may support the observed decrease in lipid peroxidation. Another possibility would be OH^•^ neutralization by atom transfer. Thus, considering the *S. tuberculata* scavenger effects on DPPH^•^ and ABTS^•^ radicals, we may suggest that the OH^•^ radical scavenging potential of extracts interfered in the oxidation process.

In conclusion, *S. tuberculata* presented different classes of metabolites, predominantly phytoecdysteroids, which, together with polyphenols, may be involved with antioxidant activity. In addition, 20E showed an *in silico* low risk, and crude extracts had very low cytotoxicity against *A. salina* larvae. Thus, due the medicinal potential revealed by *S. tuberculata*, it is important to conduct further *in vivo* studies, as well as to consider 20E as a promising molecule for further investigations on oxidative stress from a nutraceutical source.
